# Technical considerations of a game-theoretical approach for lesion symptom mapping

**DOI:** 10.1186/s12868-016-0275-6

**Published:** 2016-06-27

**Authors:** Melissa Zavaglia, Nils D. Forkert, Bastian Cheng, Christian Gerloff, Götz Thomalla, Claus C. Hilgetag

**Affiliations:** Department of Computational Neuroscience, University Medical Center Eppendorf, Hamburg University, Martinistraße 52, 20246 Hamburg, Germany; School of Engineering and Science, Jacobs University Bremen, Campus Ring 1, 28759 Bremen, Germany; Department of Radiology and Hotchkiss Brain Institute, University of Calgary, 3330 Hospital Drive NW, Calgary, AB T2N 4N1 Canada; Department of Neurology, University Medical Center Eppendorf, Hamburg University, Martinistraße 52, 20246 Hamburg, Germany; Department of Health Sciences, Boston University, 635 Commonwealth Ave., Boston, MA 02215 USA

**Keywords:** Brain lesions, Multi-perturbation Shapley value analysis (MSA), Game theory, Lesion inference, Functional prediction

## Abstract

**Background:**

Various strategies have been used for inferring brain functions from stroke lesions. We explored a new mathematical approach based on game theory, the so-called multi-perturbation Shapley value analysis (MSA), to assess causal function localizations and interactions from multiple perturbation data. We applied MSA to a dataset composed of lesion patterns of 148 acute stroke patients and their National Institutes of Health Stroke Scale (NIHSS) scores, to systematically investigate the influence of different parameter settings on the outcomes of the approach. Specifically, we investigated aspects of MSA methodology including the choice of the predictor algorithm (typology and kernel functions), training dataset (original versus binary), as well as the influence of lesion thresholds. We assessed the suitability of MSA for processing real clinical lesion data and established the central parameters for this analysis.

**Results:**

We derived general recommendations for the analysis of clinical datasets by MSA and showed that, for the studied dataset, the best approach was to use a linear-kernel support vector machine predictor, trained with a binary training dataset, where the binarization was implemented through a median threshold of lesion size for each region. We demonstrated that the results obtained with different MSA variants lead to almost identical results as the basic MSA.

**Conclusions:**

MSA is a feasible approach for the multivariate lesion analysis of clinical stroke data. Informed choices need to be made to set parameters that may affect the analysis outcome.

## Background

Lesion analysis reveals causal contributions of brain regions to mental functions, aiding the understanding of normal brain function and rehabilitation of brain-damaged patients. Historically, brain lesions were one of the few available sources of information by which functions of the human brain could be inferred. Although lesion inferences have made an enormous contribution to understanding the human brain and have laid the basis for attributing mental functions to specific brain regions [e.g. 1], they also have several drawbacks, such as the difficulty to infer function on the basis of the behavior of individual patients, the principal assumption of the localization of function, as well as the plasticity of the injured brain [2]. Today, a broad range of additional techniques exist to investigate the functions of the living brain through the correlation of behavior and cognition with brain activity, as shown by electrophysiology and functional imaging. In this context, lesion inferences, which link behavioral functions directly and causally to a neural substrate, still have an important role in modern neuroscience [2].

Despite the exciting promise of lesion inferences for identifying causal functional contributions, they are limited by several conceptual difficulties. Young et al. [3] discussed the potential unreliability of classical inference methods, such as single and double dissociations. In a single dissociation, a lesion of brain structure *a* disrupts function *A* but not function *B*; suggesting that functions *A* and *B* have some independence. Double dissociations arise when function *A* is disturbed by lesion of *a* and not of region *b*, while function *B* is disturbed by lesioning *b* and not *a*. This observation also leads to the conclusion of independent functions *A* and *B*, and their attribution to lesioned regions *a* and *b*, respectively [4]. The principal problem of single dissociations is the impossibility to assess if an apparently specific deficit arises from the impairment of a specific, localized process or from more general lesion impairment. In fact, a behavioral deficit after a lesion could be evidence for an interdependent hierarchy of functions in which the lesioned region plays a contributing role, rather than evidence for a localization of the function. Although double dissociations appear to represent a conceptual improvement over single dissociations in correctly ascribing functions to brain regions, they can also result in incorrect conclusions. Striking examples in this respect are so-called *paradoxical lesion effects*, such as the reversal of visual hemineglect during bilateral cortical or collicular inactivation in the cat brain [5]. An extensive variety of paradoxical lesion effects has been documented [6, 7], including two major types of paradoxical functional facilitation (*PFF*): *restorative PFF* and *enhancing PFF*. Restorative PFF arises when damage to intact brain tissue returns a previously sub-normal level of functioning back to normal. One example is the *Sprague effect* [8], where superior collicular lesions can result in a (partial) restoration of visual functioning following an initial visual cortical lesion of the contralateral hemisphere. By contrast, an enhancing PFF occurs when a subject with apparent nervous system pathology or sensory loss performs better than healthy control subjects on a particular task. These paradoxical effects arise because the brain is not just a collection of independent functional processors, but a complex system in which brain function emerges from the multiple interactions of distributed yet interlinked brain regions. Therefore, the conceptual problems linked to single or double dissociations also extend to higher-order inferences (e.g., triple dissociation), and it becomes apparent that, in a strict sense, the true causal contributions of brain regions to behavior can only be correctly inferred from evaluating *all* combinations of intact and injured brain regions together with their behavioral scores.

In this context, a number of traditional strategies as well as current approaches for lesion inference, all computed on a voxel-by-voxel basis, were reviewed by Rorden and Karnath [2]; such as voxel-based morphometry (*VBM*) [9, 10], *BrainVox* [11], voxel-based lesion-symptom mapping (*VLSM*) [12], and voxel-based analysis of lesions (*VAL*) [13]. Kinkingnéhun et al. [14] introduced a method called anatomo-clinical overlapping maps (*AnaCOM*) which uses, in contrast to tradition statistical approaches for voxel-wise lesion behavior mapping (*LBM*, [15]), neurologically healthy individuals as control population instead of data from neurological patients [14, 15]. All statistical approaches mentioned above have, as a main drawback, the need to control for false positives [16]. Recently, Smith et al. [17] introduced a new approach, called multivariate pattern analysis (*MVPA*), to predict the presence or absence of spatial neglect from brain injury maps. Specifically, the authors used two machine-learning techniques, based on linear and nonlinear support vector machines (*SVMs*), to classify individuals based on structural brain scans with right hemisphere lesions. A recent study of ischemic stroke patients by Forkert et al. [18] demonstrated that information about stroke location (specifically, lesion overlap measurements) can improve the prediction of functional outcome (as measured by the modified Rankin Scale) by multiclass SVMs.

Principally, lesion inference approaches can be classified on the basis of the univariate or multivariate nature of the used method. Multivariate analysis methods account for inherent dependencies among regions of interest. Such dependencies may lead to substantial mis-inferences of univariate analysis methods, as demonstrated by Mah et al. [19] through ground-truth lesion simulations. The majority of lesion mapping approaches to date has applied univariate regression models [20]. Multivariate approaches based on machine learning, such as MVPA, were introduced to lesion analyses by the work of Smith et al. [17]. Similarly, Zhang et al. [21] developed a multivariate lesion symptom mapping approach using a machine learning-based multivariate regression algorithm. The authors showed that, in comparison with VLSM, the new approach has higher sensitivity for identifying the lesion-behavior relations, both on synthetic and real datasets. A recent study by Corbetta et al. [22] proposed a multivariate approach based on machine learning to examine lesion-behavior relationships across multiple domains in a large sample of patients.

In contrast to these approaches founded on machine learning, multi-perturbation Shapley value analysis (*MSA*) [23] has been suggested as an alternative inference approach based on game theory [24]. MSA represents a mathematical method to assess causal function localization from multiple perturbation data. It considers brain regions as network elements or players that interact in a game to achieve a particular behavior. The MSA computes the contributions of these elements and also their interactions from a dataset of multiple lesions. The MSA approach has been applied to neuroscience [25, 26], biochemistry, and genetics [27, 28]. In the context of clinical lesion analysis, Kaufman et al. [29] applied MSA to lesion data and line bisection test scores of 23 right hemisphere stroke patients. The study focused on 11 grey and white matter regions and used a predictor (specifically, a k-nearest neighbor algorithm) trained on the patient injury data to obtain the line bisection performance for all injury configurations. The approach revealed that among the 11 regions of interest, only four (the supramarginal and angular gyri of the inferior parietal lobule, the superior parietal lobule, the anterior part of the temporo-parietal junction, and the thalamus) had a pivotal contributing role for the given task. While this proof-of-principle paper demonstrated the practical applicability of MSA to clinical image data, it was based on a small sample of 23 cases and low-resolution CT images. In a recent paper by our group [24], we applied the MSA approach, in comparison with other lesion inference methods, to a large multi-centre dataset of stroke patient data, to investigate functional contributions of eight large-scale bilateral volumes of interest (*VOI*s). The dataset comprised lesion patterns of 148 acute stroke patients together with their neurological deficits, assessed by the National Institutes of Health Stroke Scale (*NIHSS*, [30]). The analysis, which revealed contributions to essential behavioral and cognitive functions particularly by subcortical structures, contributed to the interpretation of NIHSS in clinical practice as well as clinical trials.

MSA is a novel computational method, which has not yet been explored in sufficient detail from a technical perspective for the application to stroke lesion inference. In the present study, we applied MSA to the same large sample of patient lesion data used previously [24], to investigate open methodological and technical aspects of the MSA approach in lesion inference and systematically determine the influence of different parameter settings on the results of this approach. More precisely, the main goals of this study were to, first, compute a sensitivity analysis on the parameters characterizing the preparation of the dataset for MSA, that is, lesion definition and lesion prediction, second, identify the most important parameters for this analysis, third, investigate MSA methodological variants and, finally, assess the suitability of MSA for processing real clinical lesion data.

## Methods

### Behavioral and lesion image data

For the present study, we used a large multi-centre dataset of stroke patients, described in a recent paper by our group [24]. The used data comprise a population of acute stroke patients included in a multi-centre observational study, designed to analyze the combined use of FLAIR (fluid attenuated inversion recovery MR imaging) and DWI (diffusion-weighted MR imaging) for identifying patients with acute ischemic stroke within 4.5 h of symptom onset (the PRE-FLAIR study [31, 32]). All patients in this study were studied within 12 h of witnessed stroke onset and severity of neurologic deficit on admission was assessed using the global NIHSS. The NIHSS, which is a rating scale resulting from a standardized neurological examination, quantifies symptom severity in acute stroke [30] by scoring 11 items representing specific abilities, with scores ranging between 0 (no symptoms, correct performance of task) and 2–4 (maximum symptom severity for corresponding item). These items include the: Level of Consciousness, Horizontal Eye Movement, Visual field, Facial Palsy, Motor Arm, Motor Leg, Limb Ataxia, Sensory, Language (Aphasia), Dysarthria, Extinction and Inattention. Higher NIHSS scores indicate a more severe impairment. A global score is calculated by summing up the individual score values. Details of imaging parameters and clinical characteristics for this study cohort were described previously [24].

### Lesion image processing

For quantitative lesion analysis, the same processing pipeline was used as described in [24]. Briefly, the lesions were segmented in each DWI dataset using a semi-automatic intensity-based method. After lesion definition, the 152 MNI brain atlas [33] was registered to each DWI dataset using a surface-based registration method. The resulting transformation was then used to transform the corresponding MNI atlas brain regions to each DWI dataset, which were then used for lesion overlap quantification (in %). The study focused on eight bilateral VOIs, defined by the MNI structural atlas: caudate (*CAU*), insula (*IN*), frontal (*FR*), occipital (*OCC*), parietal (*PAR*) and temporal lobes (*TEM*), as well as putamen (*PUT*), and thalamus (*TH*), which covered the whole brain. The overlap (in %) between each of the transformed 16 anatomical structural regions as defined in the MNI structural atlas and the patient-specific acute ischemic stroke lesion was calculated for each patient. The resulting dataset was composed of 148 patient cases with different patterns of lesioned VOIs (77 left-only, 72 right-only, one patient without lesions across all VOIs who was included in both hemisphere sets) and the corresponding global NIHSS values of the patients.

### Preliminary analysis

As in Zavaglia et al. [24], we first employed two simple approaches of lesion inference to assess the relative lesion size and frequency, using *Lesion Overlap* and *Median VOI Lesion Overlap*. The first method simply calculates the percentage of patients who display a lesion in each voxel of the MNI brain atlas (in %). The second method is similar to the first one, but is applied to VOIs instead of single voxels. This approach shows the median overlap between the 16 anatomical MNI brain atlas regions and the patient-specific acute ischemic stroke lesions. For further details see Zavaglia et al. [24].

### Multi-perturbation Shapley value analysis: general approach

As an alternative to traditional inference approaches, the MSA is a rigorous method for assessing causal function localization from multiple perturbation data. It addresses the challenge of defining and calculating the contributions of network elements from a dataset of multiple lesions (multiple perturbation experiments) and their performance scores. It objectively quantifies not only the contributions of network elements, but also their interactions. MSA is based on coalitional game theory [34]. In general, the linked system elements (in this study, the volumes of interest) can be seen as players in a game, and a *perturbation configuration* represents a subset of elements, which are perturbed concomitantly. The set of elements, which are left intact, represents the *coalition* of players. For each configuration, the performance of the system (here, the inverse of the NIHSS), which can be seen as the *worth* of the coalition, is measured. The aim of the analysis is to assign values that represent the elements’ contribution, or importance, for the overall function. The contribution of an element represents the worth of the coalition which contains the element, in relation to the worth of coalitions that do not contain it. The formal procedure is described below.

If we consider a system composed of *N* = {1, … , *n*} elements that perform a task, we can define a coalition *S*, where *S* ⊆ *N*, and a *performance scorev*(*S*), which is a real number representing the performance measured for the perturbation configuration in which all the elements in *S* are intact and the rest perturbed. The definite value in game theory and economics for this type of coalition game is the Shapley value [34]. The *marginal importance* of player *i* to a coalition *S*, with *i* ∉ *S*, is represented in Eq. 1

1 $$\Delta_{i} \left( S \right) = v(S \cup \left\{ i \right\}) - v\left( S \right)$$

The Shapley value of each player *i* ∈ *N* is defined by Eq. 2 where ${{\mathcal{R}}}$ is the set of all *n*! orderings of *N* and *S*_*i*_(*R*) is the set of players preceding *i* in the ordering *R*. If we assume that all the players are arranged in some order, all orders being equally likely, the Shapley value can be interpreted as the marginal importance of a player *i* to the set of players that precede him. We define a configuration to be an indicator vector for the set of unperturbed elements, that is a binary vector of length *n*, with *c*_*i*_ = 1 if *i* ∈ *S* or *c*_*i*_ = 0 if *i* ∉ *S*. For a detailed, formal description of MSA see [23].

2 $$\gamma_{i} \left( {N,v} \right) = \frac{1}{n!}\mathop \sum \limits_{{R \in {\mathcal{R}}}} \Delta_{i} \left( {S_{i} \left( R \right)} \right)$$

When all possible 2^*n*^ perturbation configurations are known, the Shapley value can be computed either using Eq. 2 where the summation runs over all *n*! ordering of *N,* or it can be computed as a summation over all 2^*n*^ configurations, properly weighted by the number of possible ordering of the elements (*Full Information MSA*).

### Multi-perturbation Shapley value analysis: method variants

#### Predicted MSA

Frequently, the complete set of performance scores for all combinations of the binary states of a set of regions required for MSA is not available, due to the difficulty of experimentally accessing all perturbation configurations. In those cases, a prediction model trained on the available set of configurations and performance scores can be used to predict the performance scores corresponding to all binary configurations.

In this study, a total of 2^*n*^ = 256 *binary lesion configurations* existed (where each VOI can be lesioned, “0”, or intact, “1”, and *n* = 8 is the number of VOIs for each hemisphere) and correspondingly, 256 performance scores were required for the MSA (see “Multi-perturbation Shapley value analysis: general approach” section). However, the original input dataset used in this work was composed of only 77 graded lesion configurations (describing % lesion overlap) and corresponding performance scores for the eight left VOIs and 72 graded lesion configurations and corresponding performance scores for the eight right VOIs. Therefore, we used a machine learning model trained on the available input dataset (see “Multi-perturbation Shapley value analysis: prediction of unknown performance scores” section for details) to predict the performance scores of all possible 2^*n*^ = 256 binary lesion configurations. After the prediction, all performance scores corresponding to the required 2^*n*^ binary configurations for each hemisphere were available, and it was possible to compute the Shapley value with the full information MSA (*predicted MSA*).

#### Estimated MSA

In studies where the number of system elements is too large to enumerate all configurations in a straightforward manner, the MSA can alternatively sample orderings and calculate an unbiased estimator of the contribution of each element (*estimated MSA*) as shown in Eq. 3, where ${\hat{\mathcal{R}}}$ represents a randomly sampled set of permutations.

3 $$\hat{\gamma }_{i} \left( {N,v} \right) = \frac{1}{{\left| {{\hat{\mathcal{R}}} } \right|}}\mathop \sum \limits_{{R \in {\hat{\mathcal{R}}} }} \Delta_{i} \left( {S_{i} \left( R \right)} \right)$$

It is important to note that a perturbation configuration may appear in different permutations. Therefore, the number of new configurations (and corresponding performance scores) for each sampled permutation decreases as more permutations are sampled [26]. The multi-perturbation configurations that are used in the estimated Shapley value method depend on the sampled orderings. However, the available dataset of performance measures for some set of perturbation configurations does not necessarily match the ones corresponding to a random permutation-configuration sample. In this case, as for the predicted MSA, a prediction model is trained using the available given set of perturbation configurations and corresponding performance scores and it is used to predict the performances for any perturbation configuration generated by the sampled permutations (estimated MSA). In studies where the number of VOIs (*n*) is relatively small, there is no considerable advantage in using the estimated MSA, but where the number of VOIs is much larger, its use becomes fundamental, because the number of configurations and corresponding performance scores generated by the sampled permutations is much smaller than 2^*n*^. The estimated MSA approach is suitable for large networks consisting of up to 100 network elements [26].

### Multi-perturbation Shapley value analysis: prediction of unknown performance scores

#### Choice of machine learning predictor

There is no pre-specified prediction model for MSA, and it is an important task to select the best-suited algorithm for predicting unknown performance scores on the basis of the specific configuration of available performance values. This aspect is important, because it may affect the results of the subsequent MSA analysis. Among a large number of available classifiers, one can consider, for example, naive Bayes classifiers, regression trees, nearest neighbor algorithms, random forests, and support vector machines (SVM).

The k-nearest neighbor (k-*NN*) approach has been used before for a similar purpose by Kaufman et al. [29], who applied it in their MSA study on spatial neglect patients. The k-NN method is a relatively simple interpolation algorithm in which an object is assigned a value based on the classes (i.e., functional performance score) of its *k* nearest neighbors, for instance, based on Euclidean distance. Alternatively, support vector machines have been found to be very powerful, especially in case of non-linear classification problems. Support vector machines can be applied to classification or regression problems [35]. Specifically, a supervised learning algorithm infers a function from labeled training data consisting of a set of training examples. The inferred function can be used for mapping new examples; that is, the algorithm can determine the class labels for unseen instances. In the present approach, we predicted performance scores space using the multiclass SVM (where the number of categories is larger than two) implemented in LIBSVM [36]. A sensitivity analysis on the settings of the SVM (i.e., its kernel function) was performed to select the best-suited parameters for our dataset.

#### Original-graded versus thresholded-binary dataset

Two options were investigated to predict the performance of the 256 binary configurations, using either the *original*-*graded dataset* or alternatively a *thresholded*-*binary dataset* (where “0” is lesioned and “1” is intact, created from the graded dataset after thresholding) as training dataset. Both strategies have their inherent advantages and drawbacks. On the one hand, using the original-graded dataset utilizes the information of the original data in the training without information loss due to binarization. However, the training and test data do not have the same features, since the predictor is trained with graded data and then tested with binary data (256 binary lesion configurations required by the MSA). On the other hand, using a binary dataset (after thresholding) for training, the type of training and test data is identical, at the cost of major drawbacks: the necessary choice of an arbitrary threshold for the binarization, and the consequent loss of information. Particularly, after binarization, the number of unique configurations changes, because some graded configurations become equal to each other at a binary level, but have different associated behavioral scores and also different graded lesion patterns.

The binarization threshold may be *global*, that is, using a unique value for all VOIs, or *individual*, that is, computed individually for each VOI. In our study, we explored both approaches. As a first alternative, we binarized the original-graded dataset at different global thresholds by defining each VOI as lesioned (“0”) or intact (“1”) depending on whether relative lesion size was larger or smaller than a given global threshold. Using a leave-one-out cross validation scheme, we trained the SVM respectively with the original-graded dataset graded from 0 to 1, where “0” represents a complete lesion and “1” a completely intact region (since the original overlap values are defined opposite we had to calculate values as 1—*original*-*graded dataset*), and with the corresponding thresholded-binary dataset at different thresholds. Each SVM model was evaluated with leave-one-out cross validation, using the thresholded-binary dataset obtained with different thresholds. In this way, for each binarization threshold and for both types of training dataset (original-graded or thresholded-binary), we computed the root mean squared error (*RMSE*) in terms of the difference between the real and the predicted performance score. We focused on the SVM variants with the lowest RMSE for both types of training. In turn, the same analysis was repeated for three individual thresholds, computed respectively as the first (0.25), second (0.5, median threshold) and third (0.75) quartile of the non-zero relative lesion size individually for each (individual) VOI. For the individual thresholds, we also computed a measure of *accuracy*. Specifically, we considered the prediction successful when the absolute value of the difference between the predicted and the real performance score was not larger than a maximum tolerance error (=3).

## Results

### Lesion definition

#### Lesion size and NIHSS

**Fig. 1:**
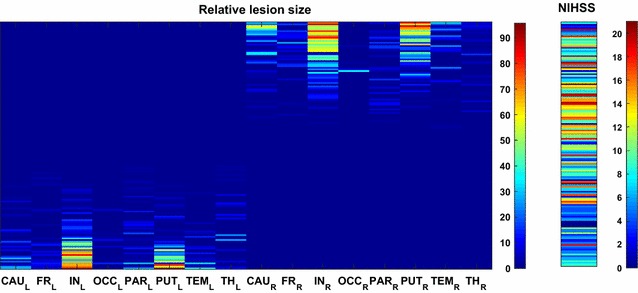
Percentage of lesioned voxels in 16 VOIs, and associated global NIHSS values for 148 patients. The *color scales* indicate the range of lesioned voxels (graded from 0 to 100%) and the range of NIHSS values (graded from 0 to 21)

Figure 1 represents the relative lesion size and the associated global NIHSS together with color bars indicating the range of variation in lesion size and NIHSS, respectively. The NIHSS is graded from 0 to 21, where zero means that the patient shows no behavioral deficit and a score of 21 indicates the severest impairment (the range is given for the current sample; higher NIHSS scores are possible). The figure demonstrates the segregation into left- and right-hemispheric lesions and the correlation of lesion size with NIHSS. Large structures, such as the cortical lobes, typically only have small relative lesions, while relative lesion sizes are larger for small (subcortical) structures.

#### Lesion overlap and median VOI lesion overlap

**Fig. 2:**
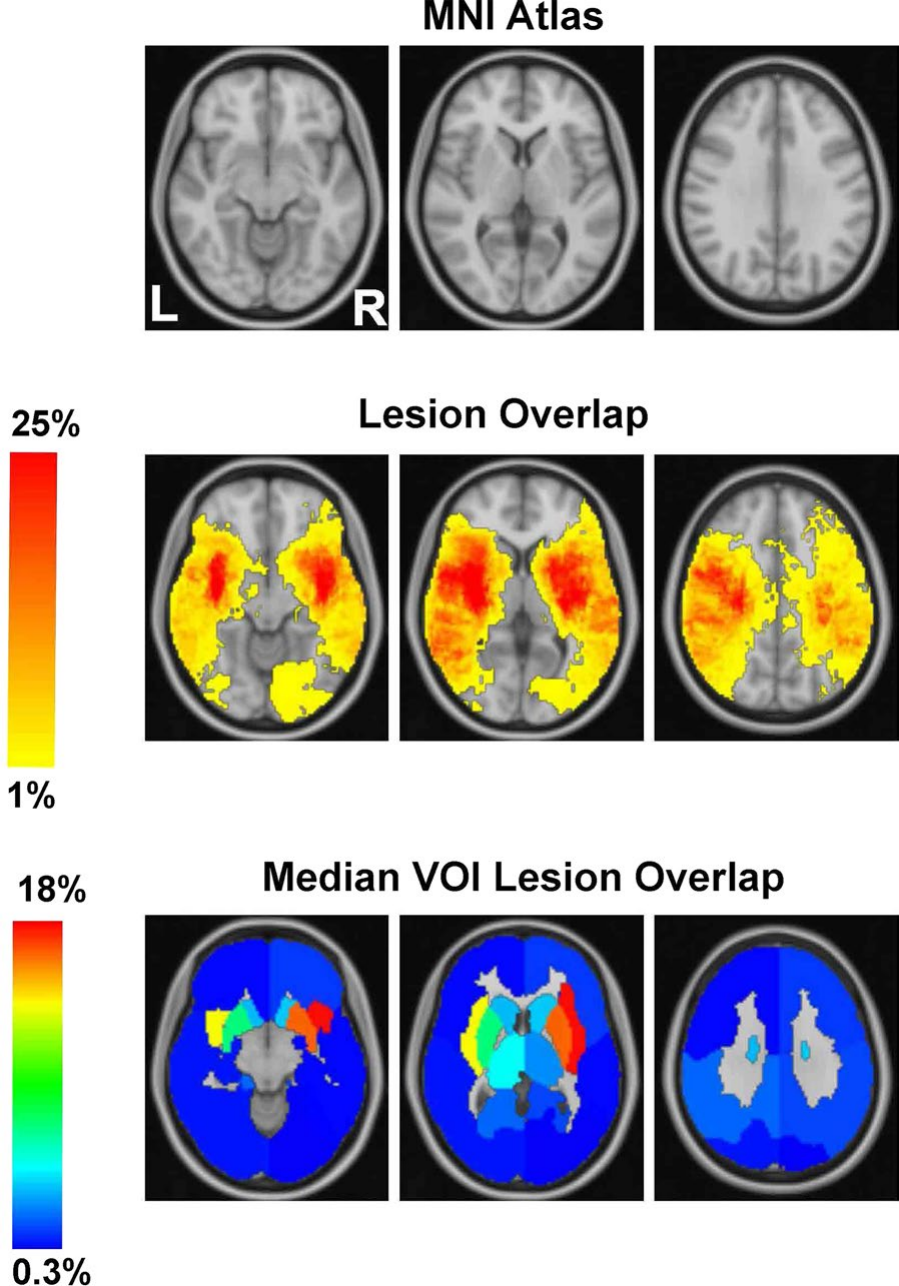
Illustration of MNI atlas (by three representative slices of the MNI atlas covering all structural regions), lesion overlap and Median VOI lesion overlap, in neurological convention. While the lesion overlap focuses at the scale of voxels, Median VOI lesion overlap shows the relative (median percentage) infarction within the confines of the predefined 2 × 8 VOIs. The *color map* is the same for all measures, but at different scales. See also [24]

**Fig. 3:**
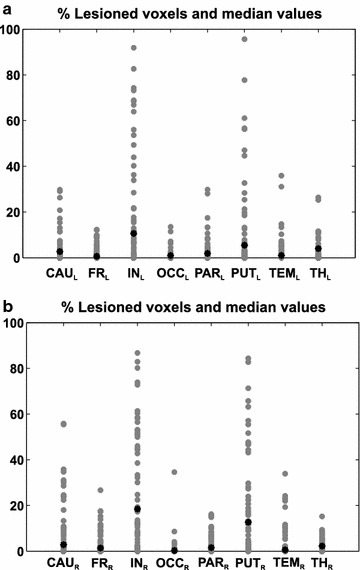
Percentage of lesioned voxels (*grey*) and median values (*black*) for each VOI in the left (**a**) and right (**b**) hemispheres respectively

Figure 2 shows the outcomes of the preliminary analysis approaches described in “Preliminary analysis” section, applied to the left and right lesions dataset separately and represented in the reference space of the MNI atlas (top row). The first, simple and straightforward assessment of the data is by the relative Lesion Overlap, shown in the second row on the MNI brain atlas (using neurological convention). This representation shows that all VOIs involved in the study are damaged to different extent. Due to these overlapping lesion patterns, it is difficult to establish a simple relationship between the lesions and the behavioral scores (NIHSS). As a second method to visualize the lesioned regions, we used Median VOI Lesion Overlap, Fig. 2, third row, showing the relative (percentage) infarction computed individually for each VOI. This method clearly indicates that, on a relative scale, the subcortical regions are most affected, especially in the right hemisphere. In Fig. 3 for each VOI in the left and right hemisphere, we also plotted the relative percentage of lesioned voxels for all patients with the corresponding median VOI infarction.

### Lesion prediction

#### Original-graded versus thresholded-binary dataset and linear- versus RBF-kernel SVM: global threshold of binarization

**Fig. 4:**
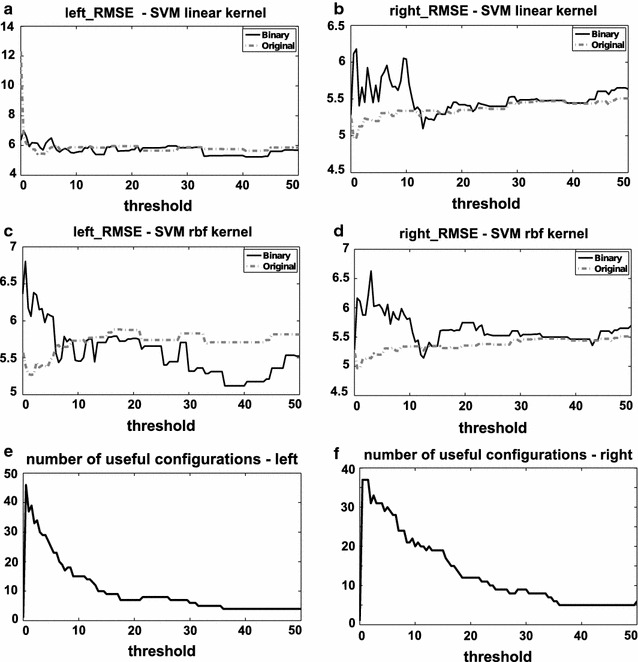
RMSE functions for SVM linear kernel (**a**, **b**) and SVM radial basis function (RBF) kernel (**c**, **d**) for both left and right hemispheres, depending on the global thresholds. Numbers of unique configurations for left and right damaged patients, depending on the global-threshold (**e**, **f**). The RMSE functions for the binary training are represented in *black*, and for the original training in *dashed grey*. The values of the RMSEs computed with the median-threshold binarization are reported here for comparison (*Left* SVM linear kernel: original-graded training RMSE = 5.5561, thresholded-binary training RMSE = 5.6454; SVM RBF kernel: original-graded training RMSE = 5.344, thresholded-binary training RMSE = 5.9467. *Right* SVM linear kernel: original-graded training RMSE = 5.3307, thresholded-binary training RMSE = 5.9663; SVM RBF kernel: original-graded training RMSE = 5.1841, thresholded-binary training RMSE = 5.8949)

In order to assess the drawbacks and advantages of the two types of training sets, we present both prediction options with the corresponding results. In Fig. 4, we show the changes of RMSEs for left and right VOIs separately, obtained with thresholded-binary and original-graded training for two SVM classifiers, depending on the global threshold of binarization. Specifically we used a linear kernel (kernel type t = 0) SVM, with parameters set to default value (SVM type s = 0, cost c = 1), and a radial basis function (RBF) kernel (kernel type t = 2), with all parameters set to default value except for the gamma parameter (SVM type s = 0, cost c = 1, gamma g = 1). In panel e and f of Fig. 4, the number of unique configurations after the binarization process is illustrated. It becomes apparent that it is not straightforward to select a global threshold of binarization that gives the best results for both types of trainings, particularly because it is not sufficient to consider the RMSE, as also the numbers of unique (useful) configurations after binarization have to be taken into account (as anticipated in “Original-graded versus thresholded-binary dataset” section). A threshold of 50 %, for example, yields a good RMSE, but, at the same time, only 4 and 6 unique configurations are available for the left and right hemisphere, respectively, since at this threshold, only bilateral putamen and insula have lesioned elements. In this context, an area-specific threshold for binarization, specifically and separately tailored for each VOI despite the variation in relative lesion size, could represent a good alternative.

#### Original-graded versus thresholded-binary dataset: individual threshold of binarization

Table 1 shows the results on the RMSE and accuracy (maximum tolerance error = 3) computed with individual thresholds, for left- and right-damaged patients datasets respectively, with both types of training and with the linear-kernel SVM (for brevity, we did not report the results of the RBF-kernel SVM). The median value threshold represents a good compromise between RMSE and accuracy, for both graded and binary training, as well as the number of useful configurations. Moreover, the classification accuracies obtained with the median threshold are considerably higher than the statistical chance levels, which were computed in the same way as prediction accuracy (maximum tolerance error ±3), but instead of the predicted scores we used NIHSS values that were randomly permutated for all patients. We repeated this procedure 100 times and obtained a mean chance level accuracy of 36 % for left regions and 34 % for right regions.

**Table 1:** RMSE and accuracy (%) for SVM linear kernel prediction for both left and right hemispheres, computed with both original-graded and thresholded-binary training

RMSE-accuracy (%)	Original training		Binary training		Number of useful configurations	
	Left	Right	Left	Right	Left	Right
First quartile	6.1–57	5.3–51	6.7–32	6.4–48	42	42
Median	5.6–57	5.3–52	5.6–54	6.0–53	37	40
Third quartile	5.6–59	5.4–54	5.5–54	5.5–57	21	24

The individual thresholds used for the binarization are the first (0.25), second (0.5, median) and third (0.75) quartile of the non zero relative lesion size for each VOI

**Fig. 5:**
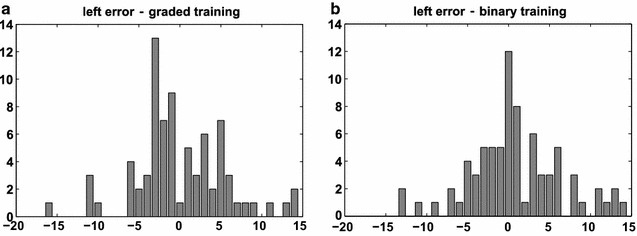
Representation of the error for left brain damaged patients computed with the leave-one-out approach with original-graded training and binary testing (**a**) and with thresholded-binary training and binary testing (**b**). The binarization is made with the median individual-threshold

**Fig. 6:**
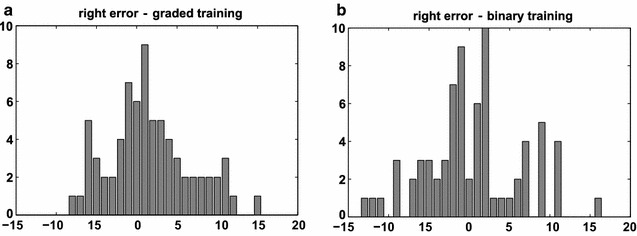
Representation of the error for right brain damaged patients computed with the leave-one-out technique with original-graded training and binary testing (**a**) and with thresholded-binary training and binary testing (**b**). The binarization is made with the median individual-threshold

**Fig. 7:**
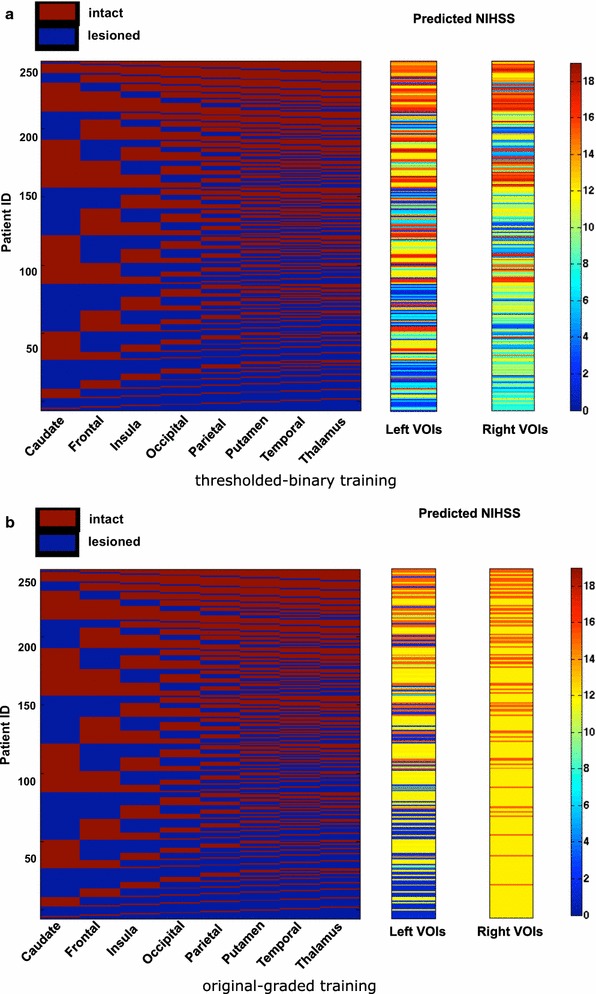
256 binary state configurations required for the MSA, sorted from all-intact (*red* 1) to all-lesioned (*blue* 0) and corresponding mean predicted scores for left and right VOIs, obtained with thresholded-binary (**a**) and original-graded (**b**) training. The *color map scale* is the same for both panels

Training of the SVM using the original-graded values instead of the thresholded-binary values intuitively promises a better prediction, due to making fuller use of the continuously defined variables. Indeed, RMSE and accuracy for graded training were slightly better than for binary training. However, this procedure incurs the problem that the training data and the predicted data are of different types (graded versus binary lesions), which leads to biases in the prediction. Specifically, the prediction of NIHSS based on graded lesion patterns led to a discontinuous spread of predicted values (cf. Fig. 7 as well as Figs. 5 and 6). While the RMSE and accuracy measures suggested that these values were within a useful range, the apparent artificial distribution of the values indicated a prediction bias. For this reason, for subsequent analyses we preferentially used a thresholded-binary dataset for the training of a linear-kernel SVM, where the binarization was implemented through a median threshold for each VOI. Figure 7 shows predicted performance scores obtained with thresholded-binary and original-graded training. Specifically, we represent the 256 binary configurations required for the MSA [sorted from all lesioned (blue = 0) to all intact (red = 1)], with the corresponding mean value of the performance scores. The performance scores were predicted with the linear-kernel SVM, for left and right VOIs, based on both thresholded-binary and original-graded dataset training and the leave-one-out cross validation. The color map range of predicted scores in the color bars is the same for panels (a) and (b). It is interesting to note that there is a substantial difference between left and right brain regions. For right regions, the predicted scores tend to vary in a smaller range compared to left ones. This observation can be ascribed to the fact that the scores are based on a battery of functions mostly associated with the left hemisphere. Moreover, it is clear that the binary training, for both hemispheres, leads to a more even spread of predicted scores, while with the original-graded training, the predicted scores tend to exhibit only a few values of the possible global NIHSS range, and the correlation between the number of intact regions and the performance score is less evident.

### MSA contributions

#### MSA: general consideration

In this section, we present the main results obtained with the MSA analysis applied separately to left and right VOIs. Specifically, we show the differences between contribution values obtained with thresholded-binary and original-graded training, between SVM classifiers (radial basis function or linear kernel), and between MSA variants (predicted MSA and estimated MSA).

#### Predicted MSA: original-graded versus thresholded-binary dataset and linear- versus RBF-kernel SVM

**Fig. 8:**
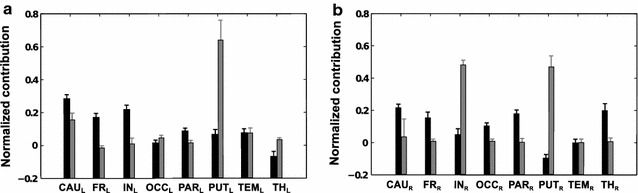
Comparison between mean contribution values obtained with original-graded (*grey*) and thresholded-binary (*black*) training, for left and right VOIs respectively, with the linear kernel SVM. Rank correlation between the contribution values of the alternative approaches is *ρ* = −0.21 (p-value = 0.62) in **a** and *ρ* = −0.05 (p-value = 0.93) in **b**

Here, we show the normalized mean MSA contribution values for the inverse NIHSS, using the linear and the RBF-kernel SVM, with thresholded-binary and original-graded training datasets, to compute unknown performance scores (predicted MSA). As left- and right-hemispheric lesions were strictly separated in the present patient sample, contributions of VOIs were computed separately for the left and right hemisphere. Standard deviation bars are derived from the leave-one-out cross validation during the prediction of performance scores. Specifically, we predicted the 256 unknown scores 77 times (for left-brain VOIs) and 72 times (for right-brain VOIs) and consequently computed the same number of MSA contribution values. Positive contribution values indicate that regions contribute positively to the performance of a task. Thus, if they are lesioned, the performance is lowered. By contrast, a negative contribution value means that the lesioning of these regions is beneficial for the performance of the task. We also show the normalized contribution values obtained with the estimated MSA, for the binary training and linear kernel SVM. In Fig. 8, we show the comparison between the contributions derived from the training with the original-graded and thresholded-binary dataset using the linear kernel SVM, for left and right VOIs respectively. For the binary training, subcortical regions, such as caudate and left insula, together with parietal and frontal lobes, were inferred to make the strongest contributions to brain functions reflected by the NIHSS. Moreover, all contributions (except for the right temporal lobe) were significantly different from zero, with negative contributions coming from right putamen and left thalamus (same results shown in [24]). Both for left and right VOIs, there were differences between the two training methods. For right VOIs the difference is more evident, especially for putamen and insula that seem to have a much stronger effect when the original-graded training is used.

**Fig. 9:**
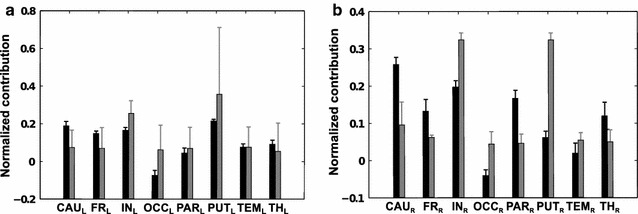
Comparison between mean contributions obtained with original-graded (*grey*) and thresholded-binary (*black*) training in left and right VOIs respectively, with the rbf kernel SVM. Rank correlation between the contribution values of the alternative approaches is *ρ* = 0.81 (p-value = 0.02) in **a** and *ρ* = 0.46 (p-value = 0.26) in **b**

In Fig. 9 we show the same quantities as Fig. 8, but generated using the radial basis function kernel SVM. The difference between the two training methods for the left VOIs is less evident than for right VOIs. Interestingly, the results obtained with the two SVM kernels do not differ considerably. We computed the rank correlation between the eight mean contribution values obtained with thresholded-binary training, with linear and RBF kernel: for left VOIs *ρ* = 0.42 (p-value = 0.30) and for right VOIs *ρ* = 0.55 (p-value = 0.17). We also computed the rank correlation between the eight mean contribution values obtained with original-graded training, with linear and RBF kernel: for left VOIs *ρ* = 0.17 (p-value = 0.7) and for right VOIs *ρ* = 0.78 (p-value = 0.03) .

#### Estimated MSA: thresholded-binary dataset and linear-kernel SVM

**Fig. 10:**
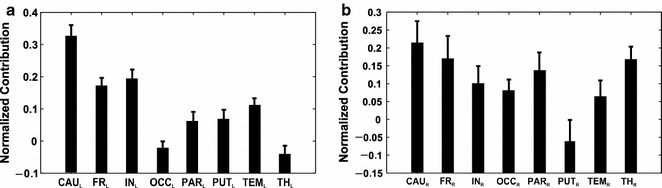
Normalized contribution values obtained with estimated MSA (100 orderings), computed with thresholded-binary training in left and right VOIs respectively, with the linear kernel SVM. Rank correlation between estimated MSA and predicted MSA (*black bars* in Fig. 8) is *ρ* = 0.98 (p-value = 0.0004) for left VOIs and *ρ* = 0.95 (p-value = 0.0011) for right VOIs

Figure 10 shows the contribution values obtained with the estimated MSA after simulations of 100 orderings of the set of multi-perturbation experiments. We used the linear kernel SVM with thresholded-binary training (individual median threshold) to predict the performance scores for all the perturbation configurations dictated by the sampled permutations. The output shows contributions which are almost identical to the contributions obtained with the predicted MSA (rank correlation is *ρ* = 0.98 for left VOIs and *ρ* = 0.95 for right VOIs, see also black contributions in Fig. 8). Also, smaller numbers of orderings (i.e., 50) work quite well (results not shown). In this study, where the number of VOIs is relatively small, there is no big advantage in using the estimated MSA, but in studies where the number of VOIs is much larger, its use becomes essential (see “Estimated MSA” section).

## Discussion

In this paper, we investigated in detail the MSA methodology and showed its application to a large dataset of stroke patients. The dataset was used in a previous study by our group [24], but here we focused on clarifying methodological and technical questions of the MSA approach in lesion inference and identifying the most important parameters for the analysis. In particular, we investigated parameters involved in the preparation of the dataset for MSA, such as the binarization threshold (global or individual) for the graded lesion dataset, the kernels of the predictor and the consequent best-suited training set for the prediction of unknown behavioral scores. We also investigated MSA methodological variants, such as the estimated MSA, that may be relevant for future studies involving more finely resolved ROIs.

### Data conditioning, choice of training set and predictor

One of the main technical limitations of the classic MSA method is the preparation of a complete lesion dataset in binary format, consisting of functional scores for all possible configurations of intact and lesioned states of VOIs, as required by the algorithm. The full information MSA approach requires complete functional information for all 2^*n*^ binary brain state configurations, where *n* is the number of regions. Thus, ideally, one would have available performance values for 2^*n*^ patients, whose lesion patterns are all different from each other. In clinical practice, this requirement is unrealistic. Even the present extensive study, which included 77 left- and 72 right-brain damaged patients, did not reach the required number of 256 distinct cases for each hemisphere, and a predictor had to be trained to obtain the full set of performance scores corresponding to the 2^*n*^ possible binary lesion configurations (predicted MSA). Moreover, in addition to the choice of the predictor (see “Choice of machine learning predictor” section) an extensive study of the best-suited training dataset is necessary. We investigated two training sets: the original-graded dataset and the thresholded-binary dataset. In clinical practice, lesion information at the VOI scale is not a binary category indicating if the region is entirely lesioned or intact, but is provided as a graded percentage of lesioned voxels of a region. In this context, it is important to note that using the original-graded dataset for training utilizes the information of the original data in the training without loss due to binarization. However, the training and test data do not have the same features, since the predictor is trained with graded data and then tested with binary data (256 binary lesion configurations required for the MSA). By using a binary dataset (after thresholding) for training, the type of training and the test data are identical, but the approach involves the choice of a threshold for the binarization, as well as the potential loss of information from thresholding. Particularly, after binarization the number of unique configurations may change, because some graded configurations become equal to each other at the binary level (i.e., these configurations are collapsed into each other), but have different associated behavioral scores and also different graded lesion patterns.

Each of the previous steps has to be carefully considered, since there is no ideal, objective method for binarizing graded data, and no predefined predictor for generating missing behavioral data. In the present study, we systematically investigated the binarization threshold and the parameters of the machine learning predictor (focusing on SVMs with linear or radial basis function kernel). In order to find the best solution for the present dataset, we performed a sensitivity analysis on global and individual thresholds for binarization and compared the results of the prediction with both original-graded training and thresholded-binary training in terms of accuracy, computed by minimizing the error in the prediction of behavioral scores. The training of the SVM using the original-graded values appeared to show a better prediction, due to the use of the continuously defined variables, as confirmed by RMSE and accuracy, which were slightly better than for thresholded-binary training. However, the use of training data of different type from the testing data led to a discontinuous spread of predicted values, as shown in Fig. 7.

The results computed with MSA by means of a thresholded-binary dataset for the training of a linear kernel SVM (threshold = median value of all non-zero percentages of lesioned voxels) showed that contributions were all significantly different from zero (with the exception of the right temporal lobe) and that subcortical regions, such as bilateral caudate, and insula, together with the parietal and frontal lobe, were inferred to make the strongest contributions to essential brain function as reflected by the NIHSS. Interestingly, MSA revealed also negative contributions, specifically from the right putamen and left thalamus (for interpretation see also [24]). The comparison of contributions value obtained with the original-graded training instead of thresholded-binary training yielded some differences, especially for the linear kernel SVM. Interestingly, the results obtained with the radial basis function SVM kernel showed no substantial differences to those obtained with linear SVM kernel, especially for right VOIs (see rank correlations in “Predicted MSA: original-graded versus thresholded-binary dataset and linear- versus RBF-kernel SVM” section).

Considering the differences using the two training methods, general recommendations can be derived. If the accuracies computed with original-graded and thresholded-binary training sets are similar and the dataset analyzed is small (i.e., composed of a small number of cases), the thresholding could cause significant loss of information due to the collapsing of configurations, and it would be preferable to choose the original-graded set to train the predictor and obtain the full set of performance scores. However, if the dataset analyzed is large, as for the data presented here, it is feasible to choose the thresholded-binary set as we did.

### MSA variants: advantages and drawbacks

The main drawback of the classic full information MSA is the need for 2^*n*^ performance scores corresponding to the binary configurations (here 256). These numbers quickly increase with the number of elements of interest, requiring, for example, 1024 configurations for ten VOIs. For this reason, the application of the standard full information predicted MSA is limited to a small number of VOIs that need to be carefully selected. The number of brain regions that can be investigated properly with the standard MSA approach depends on the available sample size, but is typically limited to around ten brain regions, given the typical sample sizes of large multi-centre stroke studies. Otherwise, the number of unknown lesion configurations and consequently the number of behavioral scores that need to be predicted grows too large, which also represents a considerable limitation for any machine learning technique. Frequently however, a resolution of about ten regions of interest provides a meaningful scope for the interpretation of lesion findings.

In this context we also investigated a variant of MSA, the estimated MSA, which is useful in studies where the number of system elements is too large to enumerate all configurations in a straightforward manner, such as studies focusing on many small VOIs or even single voxels. This MSA variant is computationally convenient, because it can sample orderings and calculate an unbiased estimator of the contribution of each element. MSA variants were already presented by Keinan et al. [26] where the authors focused on the analysis of large complex networks. Keinan et al. showed that the estimation and prediction variants successfully allowed the analysis of several neurocontrollers consisting of up to 100 neural elements. The contributions we obtained in the present study with estimated MSA were almost identical to the ones obtained with predicted MSA, and this result is encouraging in the perspective of analysing a larger number of more finely resolved anatomical or functional brain regions. In this context, it may eventually even be possible to apply the MSA for lesion inferences at the voxel-level. In doing so, it would no longer be necessary to use thresholds for binarization, since lesions at the voxel level produce binary states of lesioned and intact elementary nodes. However, such a feasibility analysis of maximum spatial resolution is beyond the scope of the present study and subject to future investigations.

### MSA versus other multivariate lesion inference approaches

How does MSA differ to other multivariate lesion inferences? Another multivariate approach is multi-area pattern prediction (*MAPP*) [24], which is based on SVM and offers a way of comparing MSA and MVPA [17] strategies. While not identical to MVPA, MAPP operates in the same spirit, by computing the leave-one-out cross-validation with n different datasets (n = number of regions), obtained respectively by removing each single region one at a time. In this way, we can measure how important each region is for the prediction procedure (i.e., by its individual contribution to the prediction error [24]). Similar to MSA, MAPP makes use of SVM in order to compute the RMSE in the leave-one-out cross-validation procedure. Like MSA, it is also applied to the thresholded-binary dataset with the corresponding performance scores, but in contrast to MSA, it does not require the full set of lesion configurations with associated performance scores.

It is also important to mention that controlling for total lesion volume can have a considerable impact on the lesion-symptom mapping approach [21]. In fact considering lesion size can be especially important to separate the specific effects of damage to a particular voxel from effects resulting from the generally higher damage likelihood in patients with large lesions compared to patients with small lesions. In this context Mirman et al. [37] reported similar anatomical results for univariate and multivariate approaches after accounting for lesion size.

An exhaustive numerical comparison between MSA and MAPP, or other recent multivariate approaches (i.e. SVR-LSM [21]), should be based on ground-truth simulations [19], which is beyond the scope of the present project.

## Conclusions

The MSA approach provides a new, principled method for the objective, multivariate computation of regional causal contributions to brain function. The approach reveals characteristic contribution patterns for behavioral and cognitive functions based on clinical scores and may provide useful guidance for rehabilitation. The method requires conditioning of the data, and we showed here that some parameters are crucial in the analysis: the threshold for binarization of graded lesion patterns, the choice of the algorithm for predicting unknown behavioral scores, and the choice of the training set. We demonstrated that there are no particular predictors or thresholds for the binarization that generally perform better than others. The choices of these settings are subjective, but we provide some general recommendations which, when considered in combination with a sensitivity analysis on these parameters, can be helpful for finding the best approach for given datasets.

In general, the results presented here are still preliminary, but indicate how MSA may allow building a matrix of causal functional contributions and provides useful guidance for rehabilitation.

## Abbreviations

MSA: multi-perturbation Shapley value analysis
NIHSS: National Institutes of Health Stroke Scale
SVM: support vector machine
VBM: voxel-based morphometry
VLSM: volume-based lesion symptom mapping
VAL: voxel-based analysis of lesions
AnaCOM: anatomo-clinical overlapping maps
LBM: lesion behavior mapping
MVPA: multivariate pattern analysis
VOI: volume of interest
FLAIR: fluid attenuated inversion recovery
DWI: diffusion weighted imaging
MRI: magnetic resonance imaging
k-NN: k-nearest neighbor
RMSE: root mean squared error
RBF: radial basis function
mRS: modified Rankin Scale
MAPP: multi-area pattern prediction
